# Antagonizing functions of BARD1 and its alternatively spliced variant BARD1δ in telomere stability

**DOI:** 10.18632/oncotarget.14068

**Published:** 2016-12-21

**Authors:** Maxim Pilyugin, Pierre-Alain André, Magdalena Ratajska, Alina Kuzniacka, Janusz Limon, Benjamin B. Tournier, Julien Colas, Geoff Laurent, Irmgard Irminger-Finger

**Affiliations:** ^1^ Department of Gynecology and Obstetrics Geneva University Hospitals, Geneva, Switzerland; ^2^ Department of Biology and Genetics, Medical University of Gdansk, Poland; ^3^ Department of Neuropsychiatry, Vulnerability Biomarkers Unit, University Hospital of Geneva, Geneva, Switzerland; ^4^ Centre for Cell Therapy and Regenerative Medicine, University of Western Australia and Institute of Respiratory Health, Nedlands, Australia; ^5^ Department of Genetic and Laboratory Medicine, Geneva University Hospitals, Geneva, Switzerland

**Keywords:** BARD1, alternative splicing, telomere alteration, shelterin, genome permutator

## Abstract

Previous reports have shown that expression of BARD1δ, a deletion-bearing isoform of BARD1, correlates with tumor aggressiveness and progression. We show that expression of BARD1δ induces cell cycle arrest *in vitro* and *in vivo* in non-malignant cells. We investigated the mechanism that leads to proliferation arrest and found that BARD1δ overexpression induced mitotic arrest with chromosome and telomere aberrations in cell cultures, in transgenic mice, and in cells from human breast and ovarian cancer patients with *BARD1* mutations. BARD1δ binds more efficiently than BARD1 to telomere binding proteins and causes their depletion from telomeres, leading to telomere and chromosomal instability. While this induces cell cycle arrest, cancer cells lacking G2/M checkpoint controls might continue to proliferate despite the BARD1δ-induced chromosomal instability. These features of BARD1δ may make it a genome permutator and a driver of continuous uncontrolled proliferation of cancer cells.

## INTRODUCTION

BARD1 has tumor suppressor functions together with BRCA1 as E3 ubiquitin ligase [1]. The BRCA1-BARD1 heterodimer ubiquitinates proteins that are involved in a number of cellular processes, including DNA repair, transcriptional regulation, chromatin remodeling, cell cycle checkpoint control, and mitosis [2–10]. BARD1 is essential for maintenance of genomic stability, as BARD1 repression in murine mammary epithelial cells caused polyploidy and chromosome instability [11]. Similarly, BARD1 knock-out mouse embryos demonstrated lethality resulting from severe impairment of cell proliferation as well as increased chromosomal aneuploidy [12].

Multiple *BARD1* mRNA isoforms of variable exon composition are expressed in human and murine cancers. Of 20 mRNAs identified in cancer tissues, at least 11 are protein coding (Supplementary Figure S1) [3, 13–19]. The full length (FL) BARD1 mRNA includes 11 exons and encodes a protein comprising an N-terminal RING-finger domain, three ankyrin repeats (ANK) and two C-terminal BRCT domains. While the RING domain is important for the BRCA1-BARD1 heterodimer formation and E3 ubiquitin ligase activity [6, 20, 21], the BRCT domains are involved in phospho-epitope binding [22, 23] and ADP-ribosylation [24]. The BARD1 C-terminus, including ANK and BRCT, has been shown to interact with a number of proteins important for carcinogenesis, such as p53 [13, 25, 26], CstF-50 [27–29], Ewing's Sarcoma oncoprotein [30], NF-kB [31], Aurora kinases [8, 32], and estrogen receptor-α [33]. It appears plausible that BARD1 isoforms of different domain composition may be involved in the same pathways as FL BARD1, yet play different roles or compete for normal BRCA1-BARD1 functions.

Further evidence for a functional link between malignant transformation and alternatively spliced BARD1 isoforms came with the identification of *BARD1* as a neuroblastoma predisposition gene in a genome wide association study. Single nucleotide polymorphisms (SNPs) in introns of *BARD1* correlated with a subclass of highly aggressive and treatment resistant neuroblastoma [34–36] and with elevated expression of the alternatively spliced BARD1β isoform [32]. *In vitro* repression of BARD1β caused SNP genotype-specific inhibition of cell proliferation in neuroblastoma cells, and overexpression of BARD1β, but not FL BARD1, led to the transformation of non-malignant fibroblasts, suggesting that BARD1β is an oncogenic driver of high-risk neuroblastoma [32].

The cellular functions of BARD1 isoforms that are associated with cancer are still unclear. There is accumulating evidence that BARD1 isoforms may antagonize the function of the BARD1-BRCA1 E3 ubiquitin ligase. In particular, BARD1β, lacking the BRCA1-interacting RING domain, binds and stabilizes the Aurora A and B kinases during mitosis, while the overexpression of either BARD1 or BRCA1 leads to degradation of the Aurora A and B kinases [8, 32], suggesting that BARD1β antagonizes this function.

BARD1δ, an isoform that lacks RING and ANK, regions critical for interaction with BRCA1 and p53, respectively [13, 25, 37–39], was found in all types of cancer investigated so far, of human and murine origin [14–19, 32], and was specifically correlated with highly aggressive clear cell ovarian cancer [14]. Interestingly, BARD1δ is as well expressed in normal human cytotrophoblasts [32, 40] and has functions as regulator of estrogen signaling [33].

Here we investigated the phenotype of BARD1δ overexpression *in vitro* and *in vivo*. We monitored cell proliferation, induction of aneuploidy and chromosome instability, as well as the stability of telomeres and the telomere-capping complex shelterin [41–44]. We suggest a molecular mechanism that explains the observed phenotype, based on novel functions of BARD1δ in telomere stability.

## RESULTS

### BARD1δ inhibits cell proliferation *in vitro*

**Figure 1:**
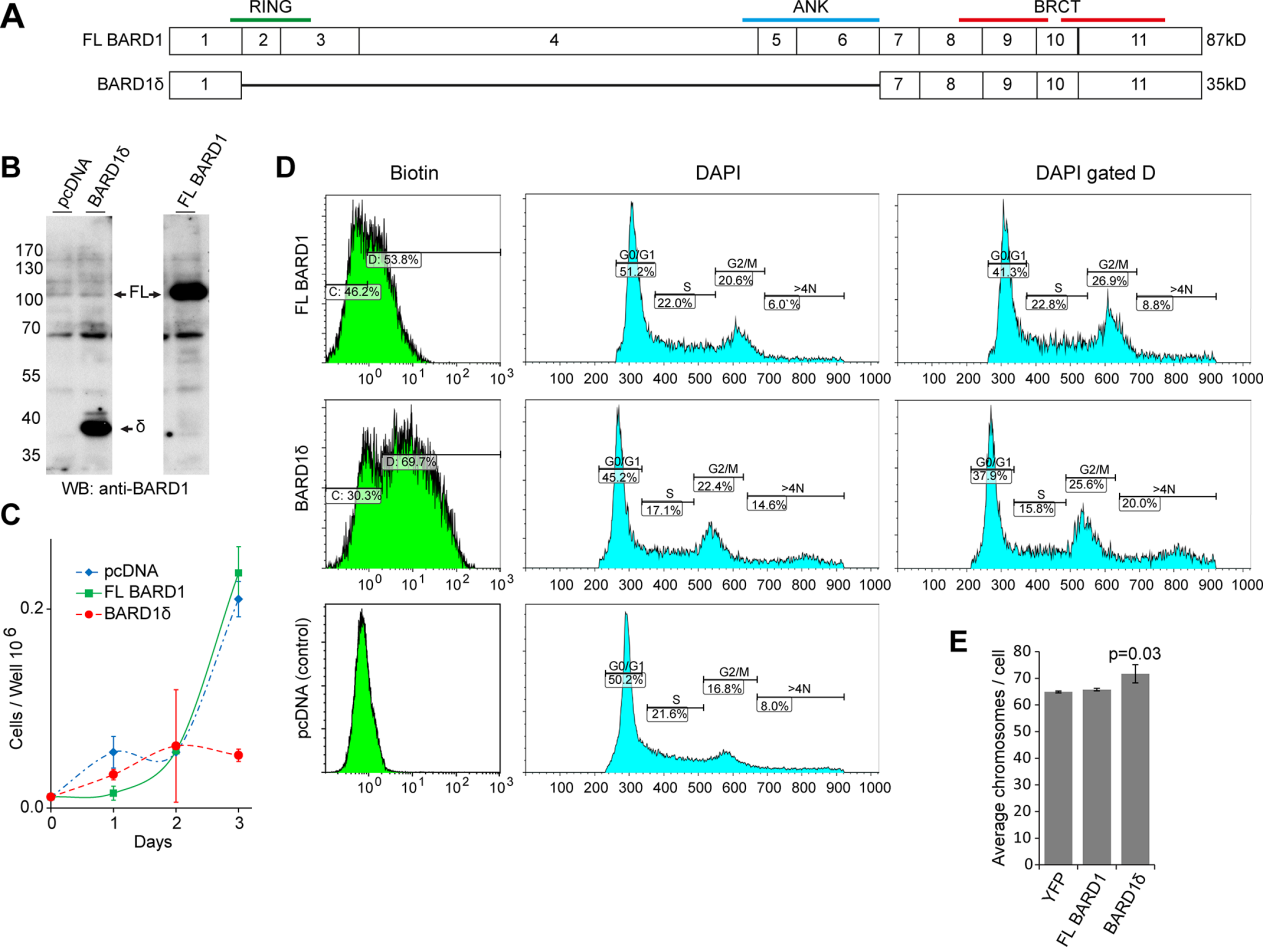
BARD1δ overexpression reduces cell proliferation rate and causes cell cycle arrest (**A**) Exon structure of FL BARD1 and isoform BARD1δ is shown with annotation of approximate positions of RING, ankyrin repeats (ANK), and BRCT domains. Predicted molecular weight is indicated on the right. (**B**) Western blots of lysates of HEK293 cells show expression of endogenous and exogenous FL BARD1 (FL) and biotin-tagged BARD1δ (δ) in cells transfected with pcDNA, FL BARD1, or BARD1δ. Western blots were probed with anti-BARD1 antibody N-19 reacting with the BARD1 N-terminus. (**C**) Growth curves of HEK293 cells transfected with pcDNA, FL BARD1, or BARD1δ. The results of three independent experiments were normalized to the cell count at day 0 and the average is presented graphically. Error bars represent standard deviation. (**D**) FACS analysis of HEK293 cells transfected with empty vector, FL BARD1-bio, or BARD1δ-bio. Biotin expression and DNA (DAPI) content of cells is presented. DNA content of 2N (S phase), 4N (G2/M), and > 4N was measured for entire sample (DAPI) and for the gated fraction of biotin expressing cells (DAPI, gated D). (**E**) The average number of chromosomes per cell in YFP, FL BARD1, or BARD1δ overexpressing HEK293 cells is presented. The error bars represent standard error of the mean (SEM). The *p-value* was defined using Student's *T-test*.

To investigate the cellular functions of BARD1δ, we transfected HEK293 cells with plasmids expressing biotin-tagged BARD1δ, biotin-tagged FL BARD1, and the empty pcDNA vector as a negative control (Figure 1A, 1B). Overexpression of BARD1δ resulted in decrease of cell proliferation by approximately 70% as compared with cells expressing FL BARD1 or control cells (Figure 1C). The growth inhibitory effect of BARD1δ was not restricted to HEK293 cells, but was also observed in other cell types (Supplementary Figure S2).

To investigate the mechanisms that caused proliferation arrest due to BARD1δ overexpression, we determined the proportion of cells in G1, S, or G2/M phase by FACS analysis of HEK293 cells expressing biotin-tagged BARD1δ or FL BARD1 (Figure 1D). We labeled the cells expressing biotin-tagged BARD1δ, FL BARD1, or empty vector with avidin and gated for the biotin-tagged cells (Figure 1D). The number of cells with S phase DNA content was reduced in the BARD1δ expressing cells, as compared to vector control or FL BARD1 expressing cells, while the number of cells with a DNA content of 4N and higher was increased by 60% in BARD1δ expressing cells.

To further confirm genomic instability associated with BARD1δ overexpression, we analyzed metaphase chromosome spreads of YFP-BARD1δ, FL BARD1, or control YFP expressing HEK293 cells. BARD1δ cells showed a statistically significant increase of chromosome numbers per cell, as compared with YFP or FL BARD1 overexpressing cells (Figure 1E). Taken together, these data suggested that elevated levels of BARD1δ inhibited the completion of mitosis and induced aneuploidy and genetic instability.

### BARD1δ blocks cell proliferation *in vivo*

**Figure 2:**
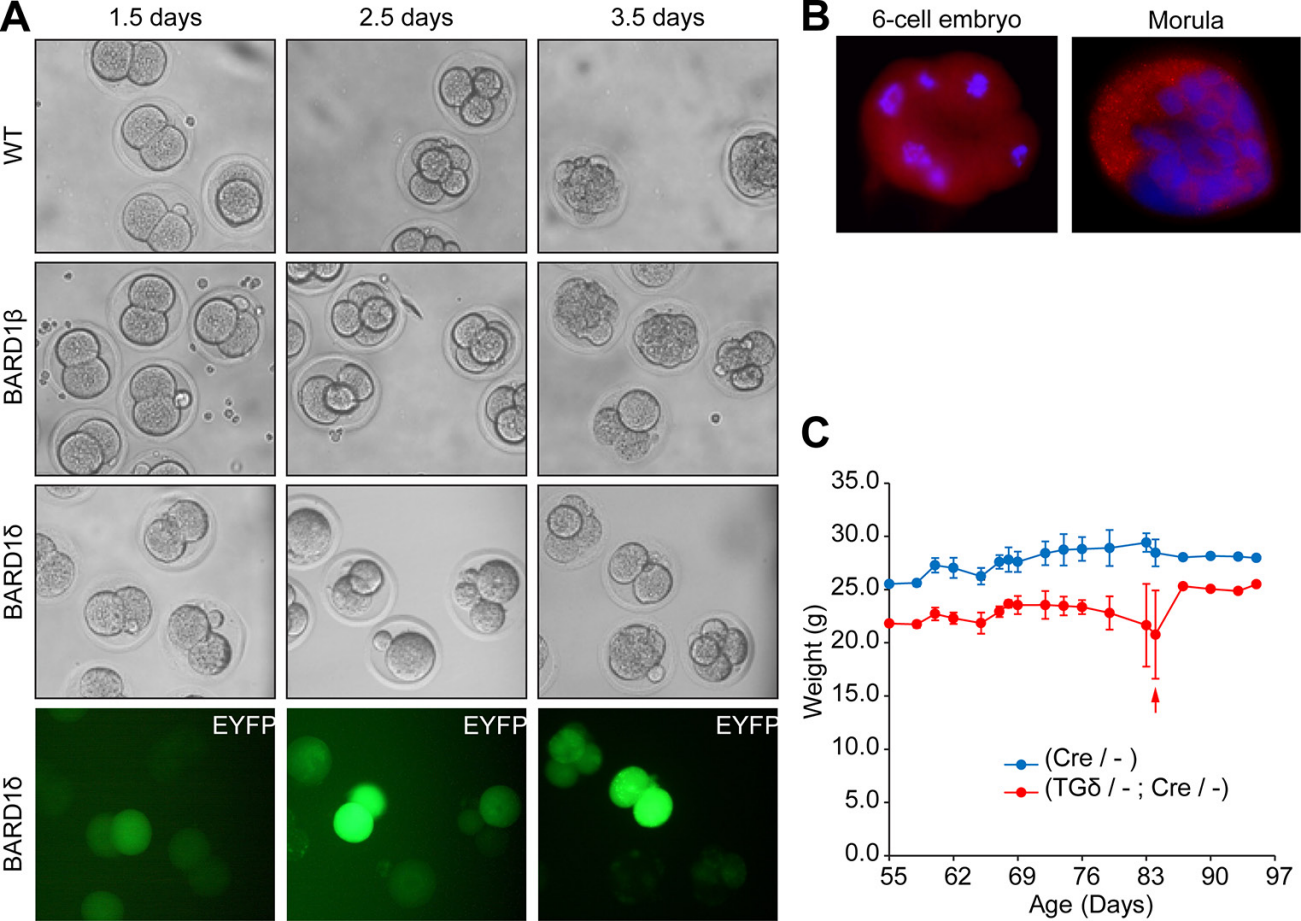
BARD1δ blocks cell proliferation in vivo (**A**) Cell divisions of fertilized oocytes after injection with BARD1β or YFP-BARD1δ (BARD1δ) transgenes. Mouse oocytes injected at the one-cell stage with control injection (WT), the YFP- BARD1β transgene, or BARD1δ (grey scale and fluorescent green), were monitored *ex-vivo* during the mouse embryonic development to the 4 and 8 cell and blastula stage after 2.5 and 3.5 days, respectively. YFP-BARD1δ injected mouse eggs showed developmental arrest at 2 or 4-cell stage after embryonic day 3.5. Experiments were performed on several consecutive days with similar results. (**B**) Immunofluorescent staining of 8-cell and morula stage wild type mouse embryos with anti-BARD1 antibody directed against exon 4 for expression of endogenous BARD1. (**C**) Weight loss of the YFP-BARD1δ expressing transgenic mice. Weight gain or loss was monitored of YFP-BARD1δ Cre (TGδ/-; Cre/-) or BL6 Cre mice (Cre/-) upon tamoxifen driven *CRE* induction. Red arrow indicates death* of one YFP-BARD1δ Cre (TGδ / -;Cre /-) animal. The error bars show the standard deviation.. *Sacrificed because of too rapid weight loss.

In an attempt to study the effect of BARD1δ on cell proliferation *in vivo*, we micro-injected YFP-tagged BARD1δ into the pronucleus of fertilized mouse eggs and monitored their development to morula and blastula stages *ex vivo* (Figure 2A). While mock injected embryos divided and developed normally, as well as the embryos injected with an expression construct for the pro-proliferative isoform BARD1β [8, 32], many of the oocytes injected with the YFP-BARD1δ expression vector were arrested at the 2 or 4-cell stage, and all arrested embryos were YFP-positive (Figure 2A).

These results confirmed a growth inhibitory function of BARD1δ in non-malignant cells *in vivo*. Further, this experiment demonstrated that constitutive expression of BARD1δ prevented embryonic development of the mouse. Previous reports showed that BARD1 was expressed during early development of the mouse (11) and it was essential for embryonic development (12). Immunofluorescence staining of wild type embryos confirmed expression of endogenous FL BARD1 at early stages (Figure 2B). Together these data suggest that FL BARD1 is required for early development, but BARD1δ might play an inhibitory role.

To study BARD1δ transgene-dependent growth inhibition at later stages, we generated conditional CRE recombinase-dependent YFP-BARD1δ mice (Supplementary Figure S3A). The YFP-BARD1δ transgene was silenced by insertion of a floxed STOP element between the CMV promoter and the translation start.

Activation of the YFP-BARD1δ transgene was achieved with tamoxifen-inducible CRE (Supplementary Figure S3B–S3D). Transgenic YFP-BARD1δ/- mice were crossed to BL6 or Cre/- mice. Upon tamoxifen induction of the CRE recombinase we observed weight loss associated with increased mortality of YFP-BARD1δ/-;Cre/- mice, in comparison with control wild type or Cre/- mice (Figure 2C). The weight loss of YFP-BARD1δ transgenic mice could be explained by and is consistent with the observed function of YFP-BARD1δ in inhibiting cell division and proliferation *in vitro* and *in vivo* (Figures 1D, 2A; Supplementary Figure S2).

Moreover, we found that the proportion of YFP-BARD1δ transgenic mice in the progeny of a cross between BL6 wild type or Cre/- mice was less than the expected 50% (Supplementary Figure S4A). We also found significantly higher early mortality of YFP-BARD1δ/- mice and even more so of YFP-BARD1δ/-;Cre/- mice (Supplementary Figure S4B). These data suggested that leaky expression of BARD1δ (Supplementary Figure S3D) might be sufficient to compromise normal embryonic development and growth of transgenic mice.

### BARD1δ overexpression causes aberrant telomere structures *in vitro* and *in vivo*

**Figure 3:**
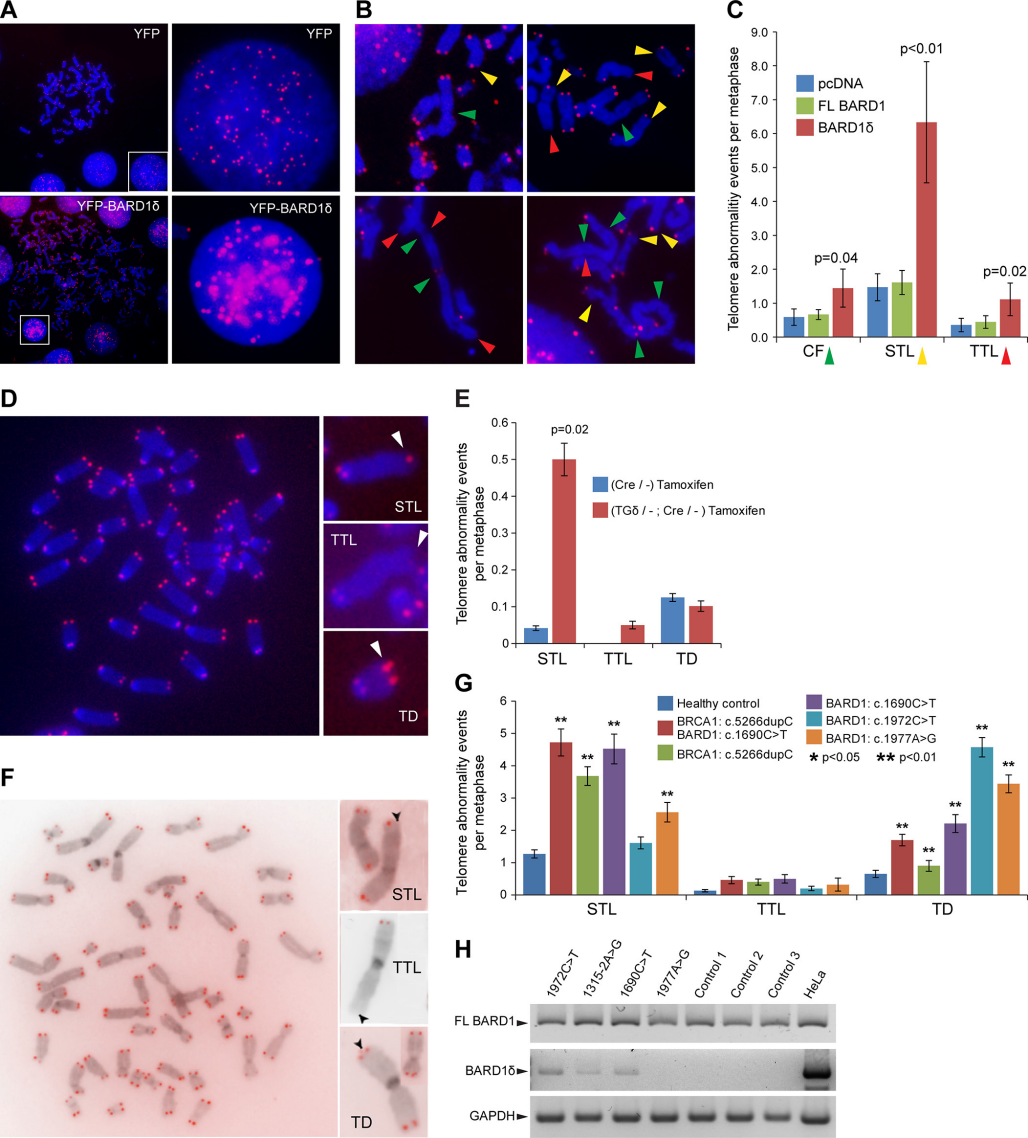
BARD1δ overexpression causes telomere aberrations in vitro and in vivo (**A**) Telomere FISH in HEK293 cells transfected with YFP or YFP-BARD1δ is shown. Metaphase spreads and interphase cells (left panel) and close-ups of interphase cells (right panel) show the distribution of telomere FISH signal in control cells (YFP) and cells transfected with YFP-BARD1δ. (**B**) Examples of telomere FISH on metaphase spreads of HEK293 cells transfected with YFP-BARD1δ show chromosome and telomere abnormalities: chromosomal fusions (CF) (green arrowheads), sister telomere loss (STL) (yellow arrowheads), and terminal telomere loss (TTL) (red arrowheads). (**C**) The frequencies of CF, STL and TTL per metaphase are shown for FL BARD1 and BARD1δ overexpressing cells. The data represent the average for at least 17 metaphase spreads. The error bars represent SEM. The *p-value* was defined using Student's *T-test*. (**D**) Example of telomere FISH staining of metaphase chromosome spreads of cultured lymphocytes from YFP-BARD1δ/-;Cre/- mice after activation of the transgene (left panel). Examples of telomere aberrations (right panel) are shown: sister telomere loss (STL), terminal telomere loss (TTL) and telomere duplication (TD). (**E**) The frequency of telomere abnormalities, STL, TTL, and TD in cultured lymphocytes from tamoxifen treated YFP-BARD1δ/-;Cre/- mice and tamoxifen treated Cre/- control mice is shown. The data represent the average of at least 24 metaphases counted for each genotype. The error bars represent SEM. The *p-value* was defined using Student's *T-test*. (**F**) Telomere FISH staining of metaphase chromosome spreads of cultured lymphocytes from human *BARD1* mutation carrier (left panel). Examples of observed telomere abnormalities are shown: sister telomere loss (STL), terminal telomere loss (TTL) and telomere duplication (TD) (right panels). (**G**) Quantification of telomere abnormalities (TTL, STL, and TD) in the cultured lymphocytes from cancer patients with germ line mutations in *BARD1* and/or BRCA1 and healthy subjects (*n* = 10). The data represent the average from at least 45 metaphases for each patient and 90 metaphases for healthy controls. The error bars represent SEM. The *p-value* was defined using Student's *T-test*. (**H**) RT-PCR specific for FL BARD1 and BARD1δ expression was performed on blood cells from *BARD1* germline mutation carriers and healthy controls. HeLa cells were used as control for amplification and RT-PCR and GAPDH for RNA quality.

The chromosome alterations observed with BARD1δ overexpression (Figure 1E) could be caused by chromosome and telomere instability [45]. We therefore investigated whether BARD1δ affected telomere integrity. We performed telomere FISH on HEK293 cells expressing exogenous YFP-BARD1δ or YFP. In interphase cells, individual telomere FISH signals were abnormally clustered in BARD1δ expressing cells, while control cells showed a regular distribution of telomere staining dots (Figure 3A). In metaphase spreads of cells overexpressing YFP-BARD1δ we observed an increased frequency of end-to-end chromosome fusions (CF), often associated with Sister telomere loss (STL) and terminal chromosome telomere loss (TTL), as compared with FL BARD1 or YFP expressing cells (Figure 3B, 3C).

To investigate the impact of BARD1δ on chromosome integrity *in vivo*, we performed telomere FISH on metaphase spreads of lymphocytes from YFP-BARD1δ/-;Cre/- mice and Cre/- mice as control, after tamoxifen treatment (Figure 3D). Cells from YFP-BARD1δ/-;Cre/- mice showed significantly higher numbers of STL than cells from the Cre/- control mice. The number of TTLs and telomere duplications (TD) was not significantly increased (Figure 3E). These results suggest that increased expression of BARD1δ induces telomere alterations *in vivo*.

### Lymphocytes from breast/ovarian cancer patients with germline mutations in BARD1 show telomere instability

As described recently, specific *BARD1* germline mutations promote expression of alternatively spliced BARD1 mRNAs and reduction of FL BARD1 mRNAs [46, 47]. We investigated the implication of these mutations in telomere integrity. We conducted telomere FISH experiments using peripheral blood lymphocytes from breast and ovarian cancer patients with germline mutations in *BARD1* or *BRCA1*, namely *BARD1* c.1690C > T, *BARD1*c.1972C > T, *BARD1*c.1977A > G, *BRCA1* c.5266dupC [48], and the *BRCA1* c.5266dupC and *BARD1* c.1690C > T double mutation (Supplementary Figure S1B), as well as from healthy control subjects. The c.1690C > T mutation is a nonsense mutation that promotes alternative splicing [47] resulting in mRNAs that encode truncated proteins lacking the BRCT domains. The c.1972C > T mutation results in an arginine to cysteine substitution located between the two BRCT domains. The c.1977A > G mutation promotes expression of a transcript lacking exons 2–9 [47]. The c.5266dupC mutation causes a frame shift in exon 20, a region that codes for the BRCT repeats in *BRCA1* [48]. Hence all these mutations lead to loss or deficiency of the BRCT domains.

We observed a significant increase of telomere alterations, namely STL, TTL, and TD, in the cells of all *BARD1* and *BRCA1* mutation carriers in comparison with cells from healthy controls (Figure 3F). The *BRCA1* and *BARD1* double mutation carrier showed the highest frequency of STL (4.7/metaphase) and TTL (0.5/metaphase). TD was most frequent in the *BARD1* mutation carriers, reaching from 2.2 to 4.5 incidents per metaphase. We observed a similar frequency of TTL in the *BRCA1* c.5266dupC carrier (0.4 TTL/ metaphase) and the *BARD1* c.1690C > T mutation carrier (0.5 TTL/ metaphase). In comparison, lymphocytes of the healthy control group (*N* = 10) showed an average frequency of 1.3 for STL, 0.1 for TTL, and 0.7 for TD per metaphase (Figure 3G).

The BARD1 germline mutations predict a reduction of FL BARD1 expression [47], which might explain the genetic instability as described before [11, 12]. Loss of FL BARD1 was frequently found associated with expression of isoforms in many cancers [16]. We performed RT-PCR to investigate the balance between FL BARD1 and BARD1δ mRNA expression in patients with *BARD1* mutations and healthy controls. We observed the expression of BARD1δ in the patients carrying the 1315-2A > G and 1690C > T mutations causing exons 5 and 8 skipping, respectively, and missense mutation 1972C > T, while we did not detect BARD1δ PCR fragments in healthy controls and in the patient with the BARD1 1977A > G silent mutation (Figure 3H). These observations suggest that either an increase of BARD1δ or reduced expression of FL BARD1 affects telomere alterations.

### BARD1δ compromises the sub-cellular localization of telomeric proteins

**Figure 4:**
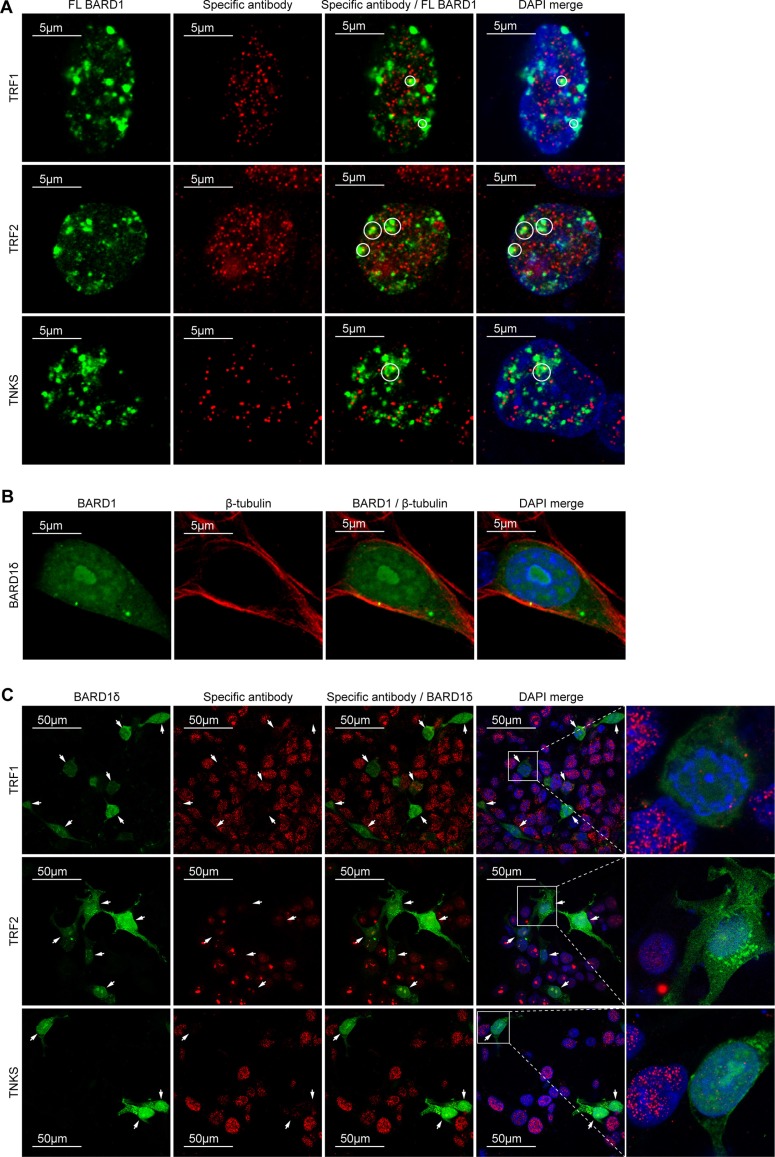
BARD1δ but not FL BARD1 affects the localization of telomere binding proteins (**A**) Confocal immunofluorescence microscopy shows the localization of biotin-tagged FL BARD1, stained with avidin (green) and antibodies against telomere binding proteins (red) in the interphase nuclei of transfected HEK293 cells. Telomere binding proteins detected are indicated at the left side. Areas of co-localization of FL BARD1 and telomere binding proteins are circled in the merge of red and green and red, green and DAPI (DAPI/merge). (**B**) Confocal immunofluorescence microscopy showing the distribution of BARD1δ-bio in transfected cells. The cells were double-stained with avidin detecting biotin (green) and anti β-tubulin antibody (red). (**C**) Confocal immunofluorescent microscopy shows the distribution of the overexpressed BARD1δ-bio (green) and telomere binding proteins (red) in the interphase nuclei of HEK293 cells transfected with BARD1δ-bio (green). Antibodies used for detection of telomere binding proteins are indicated at the left side. The cells expressing BARD1δ-bio are indicated with arrows. The right panel shows higher magnification of the boxed BARD1δ-bio expressing cells with adjacent non-transfected cells, which in comparison show high expression levels of telomere binding proteins.

To investigate the mechanism behind the telomere instability induced by BARD1δ, we performed immunofluorescence microscopy to determine the subcellular localization of BARD1δ as compared with FL BARD1. While FL BARD1 localized to nuclear dots in interphase (Figure 4A) as reported [11, 49, 50], BARD1δ was diffusely distributed in the nucleus and to a lesser extent in the cytoplasm (Figure 4B).

The localization of FL BARD1 to nuclear dots and its protective function in telomere stability led us to hypothesize that FL BARD1 might interact with the telomere complex. We therefore investigated the co-localization of FL BARD1 and BARD1δ with proteins of the telomere complex shelterin, which is essential for the maintenance of the integrity of telomeres and the prevention of chromosomal end to end fusions [41, 42, 51]. We performed immunofluorescence microscopy to determine the subcellular localization of the shelterin components TRF1, TRF2, and TNKS as a function of FL BARD1 (Figure 4A) or biotin-tagged BARD1δ (Figure 4C) overexpression. We found TRF1, TRF2, and TNKS localized to nuclear dots in the majority of non-transfected control cells and in FL BARD1 overexpressing cells. A fraction of overexpressed FL BARD1 co-localized with TRF1, TRF2, and TNKS in nuclear dots (Figure 4A). We further confirmed FL BARD1 localization to telomeres with experiments that showed co-localization of FL BARD1 with telomere repeats and the telomerase reverse transcriptase (TERT) in various cell lines (Supplementary Figure S5). These results are consistent with a function of FL BARD1 at telomeres, presumably as binding partner of BRCA1 [52, 53].

BARD1δ overexpressing cells, on the contrary, showed strongly reduced staining for TRF1, TRF2, and TNKS in nuclear dots (Figure 4C). These results suggested that BARD1δ induced their depletion from telomeres.

## FL BARD1 and BARD1δ interact with components of the shelterin complex

**Figure 5:**
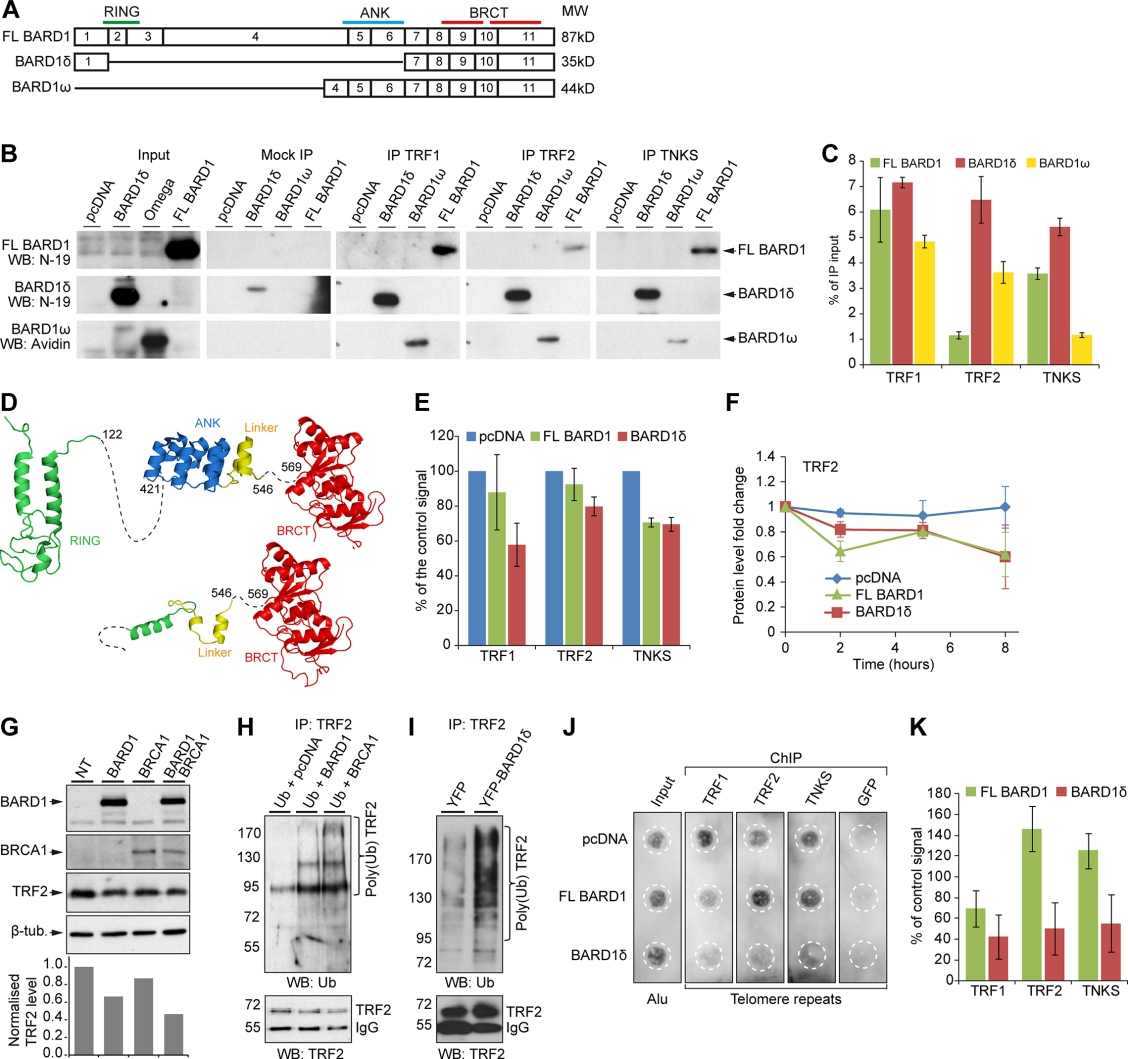
FL BARD1 and BARD1δ interact with telomere binding proteins and differentially affect their cellular concentration and localization (**A**) Exon structure and domain composition of FL BARD1, BARD1δ, and BARD1ω. (**B**) Western blot of the co-IP of overexpressed biotin-tagged FL BARD1, BARD1δ, and BARD1ω with telomere binding proteins (TRF1, TRF2, TKNS) in HEK293 cells. The protein input, mock IP (beads only), and IP with telomere binding proteins are shown. Western blots were probed with anti-BARD1 N-19 antibody to detect FL BARD1 and BARD1δ, and with HRP-conjugated avidin to detect BARD1ω. (**C**) Quantification of FL BARD1, BARD1δ, and BARD1ω co-IP with telomere binding proteins. The amount of co-precipitated BARD1 isoforms is presented as a percentage of the input protein extracts. Values correspond to the mean of three independent experiments. The error bars represent SEM. (**D**) Secondary structure models for FL BARD1 (upper) and BARD1δ (lower). RING (green) [66], ANK (blue) [67], and BRCT domains (red) [68] are indicated. “Linker” (yellow) refers to the protein sequence between ANK and BRCT domains. (**E**) The steady-state levels of telomere binding proteins of cell extracts of HEK293 cells expressing FL BARD1 or BARD1δ were measured on Western blots by densitometry. The intensity of specific bands was normalized to intensity of actin. The values of control cells transfected with empty pcDNA vector (blue bars) was taken as 100%. The error bars show SEM for two independent experiments. (**F**) Protein stability of TRF2 in HEK293 cells expressing FL BARD1 or BARD1δ after cycloheximide treatment. The protein levels in cell extracts measured by densitometry at different time points after cycloheximide treatment are shown. Signal intensities were normalized to actin intensities and to the intensity at time point zero. The error bars show SEM for two independent experiments. (**G**) TRF2 expression in HEK293 cells overexpressing FL BARD1 (BARD1), BRCA1, or FL BARD1 (BARD1) and BRCA1. Western blots were probed for TRF2 and β-tubulin expression. The chart shows the quantification of TRF2 signal in cell extracts. The intensity of TRF2 signal was normalized to β-tubulin intensity and to the signal from non-transfected (NT) control cells. (**H**) TRF2 ubiquitination in cells overexpressing FL BARD1 (BARD1) or BRCA1 was assayed. Cells transfected with HA-tagged ubiquitin and either pcDNA vector, FL BARD1, or BRCA1 expression constructs. Anti-TRF2 antibody was used for IP of TRF2. Western blots of IPs were probed with anti-ubiquitin or anti-TRF2 antibody. (**I**) TRF2 ubiquitination in the cells overexpressing YFP or YFP-BARD1δ. Anti-TRF2 IP was probed on Western blots with anti-ubiquitin or anti-TRF2 antibody. (**J**) Telomere repeat chromatin immunoprecipitation (ChIP) assay. Cells transfected with pcDNA or FL BARD1 or BARD1δ expression constructs were used for ChIP with antibodies against TRF1, TRF2, or TNKS, or GFP as negative control. The precipitated chromatin was dot-blotted and probed for telomere repeats or Alu repeats as a loading control (Input) in indicated areas (white dashed circles) and quantified. (**K**) Quantification of telomere ChIP. The intensity of the signal from telomere repeats was normalized to signals of input DNA and Alu repeats. The error bars show standard error of the mean for two independent experiments.

As FL BARD1 and not BARD1δ localized to telomeres, but BARD1δ and not FL BARD1 affected the depletion of telomere binding proteins from telomeres, we were interested in establishing whether FL BARD1 and/or BARD1δ interacted directly with components of the shelterin complex and/or affected their turnover. We first tested the interaction of FL BARD1 and BARD1δ with TRF1, TRF2 and TNKS. We expressed biotin-tagged FL BARD1, BARD1δ, and BARD1ω, an N-terminal truncated BARD1 isoform that unlike BARD1δ comprised the ANK repeats (Figure 5A), in HEK293 cells and performed co-immunoprecipitation (IP) of BARD1 proteins with antibodies raised against TRF1, TRF2, and TNKS. The co-IP was monitored on Western blots for FL BARD1, BARD1δ, or BARD1ω (Figure 5B) and control precipitation of TRF1, TRF2 and TNKS (Supplementary Figure S6A). The efficiency of each co-IP was determined by measuring the intensity of the signals of FL BARD1, BARD1δ, and BARD1ω normalized to the input signals (Figure 5C). We found that BARD1δ was co-precipitated equally well by all antibodies, while FL BARD1 and BARD1ω were less efficiently precipitated, in particular by anti-TRF2 and anti-TNKS antibodies. We performed inverse co-IPs, and BARD1δ was co-precipitated more efficiently than FL BARD1 (Supplementary Figure S6B, S6C). These results suggested that the ANK repeats, present in FL BARD1 and BARD1ω, but not in BARD1δ, had an inhibitory effect on binding.

To understand why BARD1δ showed stronger binding than FL BARD1 and BARD1ω to all tested proteins, but with a most important difference for TRF2, we mapped the TRF2 binding region on the BARD1 protein sequence (Supplementary Figure S7). TRF2 bound most efficiently to the “linker” region between the ANK and the BRCT domains, while the adjacent region comprising the ANK repeats had an inhibitory effect on binding. This is consistent with the results obtained for the co-IP experiments, and confirms that the ANK repeats have an inhibitory effect on FL BARD1 and TRF2 interaction.

Structural modeling of FL BARD1 and BARD1δ provides an explanation for the enhanced affinity of BARD1δ to the interacting proteins (Figure 5D). In BARD1δ, the “linker” helix between ANK and BRCT domains is significantly more surface exposed and less constrained than it is in FL BARD1 or BARD1ω, due to the absence of the ANK repeats. Such enhanced structural flexibility is often observed for short disordered amino acid stretches that serve as protein binding regions [54] and provides a rationale for BARD1δ's stronger binding to components of the shelterin complex than FL BARD1.

### TRF2 is a target of the BRCA1-BARD1 E3 ubiquitin ligase

As BARD1δ and FL BARD1 bound to telomere binding proteins with different affinity, we suspected that this might influence their steady-state protein concentrations or turnover. We therefore monitored the steady-state levels and degradation rates of TRF1, TRF2, and TNKS in the presence or absence of FL BARD1 or BARD1δ overexpression in HEK293 cells on Western blots. FL BARD1 as well as BARD1δ overexpression led to decreased levels of TRF1, TRF2, and TNKS (Figure 5E). We then investigated protein degradation by assaying the levels of TRF1, TRF2, and TNKS before and after blocking of protein synthesis by treatment with cycloheximide. We found that the level of TRF2 was reduced at two hours after treatment in FL BARD1 and less so in BARD1δ overexpressing cells (Figure 5F) and further reduction was observed after 8 hours for both FL BARD1 and BARD1δ, but not for control cells. At the same time, we did not observe notable decrease of the levels of TRF1, TNKS and TERT by either FL BARD1 or BARD1δ overexpression (Supplementary Figure S8).

These data might suggest that BARD1δ, ubiquitously distributed in the nucleus and cytoplasm, prevents telomeric proteins from binding to telomeres and causes their diffuse distribution and presumably degradation.

As BRCA1 and FL BARD1 have E3 ubiquitin ligase functions at telomeres [53, 55], and TRF2 degradation was increased in FL BARD1 expressing cells, we hypothesized that TRF2 might be a novel target of the BARD1-BRCA1 ubiquitin ligase. We therefore overexpressed either FL BARD1 or BRCA1, or both in HEK293 cells and monitored TRF2 concentrations (Figure 5G). TRF2 levels were decreased in cells overexpressing either FL BARD1 or BRCA1, or both. We then co-expressed HA-tagged ubiquitin with either FL BARD1 or BRCA1 to monitor TRF2 ubiquitination as a function of FL BARD1 or BRCA1 overexpression (Figure 5H). We observed the formation of ubiquitinated forms of TRF2 in cells expressing exogenous BRCA1 or FL BARD1, as compared to control cells transfected with empty vector, while levels of the non-ubiquitinated form of TRF2 were decreased in FL BARD1 or BRCA1 expressing cells. These results confirmed that the BRCA1-BARD1 E3 ubiquitin ligase induces TRF2 ubiquitination. Surprisingly, BARD1δ overexpressing cells presented increased levels of poly-ubiquitinated TRF2 (Figure 5I). These results suggest that dislocation of TRF2 from the telomeres enhances its ubiqutination by BRCA1-BARD1 and/or possibly other ubiquitin ligases [56].

### BARD1δ depletes components of the shelterin complex from the telomeres

To understand the mechanisms that lead to the telomere instability phenotype of BARD1δ overexpressing cells, we sought to determine whether BARD1δ and/or FL BARD1 influence the binding or re-localization of TRF1, TRF2, or TNKS to telomeres.

To do this, we performed chromatin immunoprecipitation (ChIP) from cells overexpressing FL BARD1 or BARD1δ, or cells transfected with the empty vector, using antibodies against TRF1, TRF2, and TNKS. An anti-GFP antibody was used as a negative ChIP control. The precipitated chromatin was dot-blotted and probed for telomere repeats, or Alu repeats as a control, and quantified (Figure 5J, 5K). In FL BARD1 expressing cells we observed a reduction of the TRF1 signal by 40%, compared to control cells, but no decrease of TRF2 or TNKS binding to telomere repeats. Cells overexpressing BARD1δ, however, showed a 60% decrease of telomere binding for all three proteins. Importantly, considering the BARD1δ transfection rate of 70% in our experiments, as determined by FACS analysis (Figure 1D), the decrease of telomere protein levels on telomere repeats could be estimated as nearly 90% in BARD1δ expressing cells. These data are consistent with and explain the observed loss of immunofluorescence staining for telomere binding proteins in nuclear dots in BARD1δ overexpressing cells (Figure 4C) and suggest that BARD1δ induces telomere instability by depleting components of the shelterin complex from the telomere.

Thus, FL BARD1 might have essential functions in the maintenance of telomere integrity, presumably with BRCA1 [57], but the interaction of BARD1δ with telomere proteins antagonizes FL BARD1 functions.

## DISCUSSION

Genomic instability is a hallmark of cancer. However, the molecular mechanisms that cause numerical or structural chromosomal aberrations in epithelial cancers are poorly understood. Altered expression of genes that control cell proliferation and the maintenance of genome stability are possible causes [58]. Chromosomal instability may even be evolutionary selected to maintain uncontrolled cell proliferation of cancer cells [59]. Thus, defects in essential cellular mechanisms such as maintenance of telomere integrity could play a role as cancer permutator by providing an opportunity for rearranging DNA and chromosomes.

This study demonstrates that an isoform of BARD1, BARD1δ, has such functions through inducing telomere instability. We show that BARD1δ overexpression leads to aneuploidy and chromosomal aberrations *in vitro*. BARD1δ overexpression causes such deleterious effects on chromosomal instability by compromising the integrity of telomere structures causing telomere attrition and telomere fusions, leading to cell cycle arrest.

Importantly we made the same observations in *in vivo* experiments. We found that BARD1δ in transgenic mice presented telomere aberrations after induction of the transgene, and embryonic development was completely blocked by constitutive expression of BARD1δ (Figure 2). Even the leaky expression BARD1δ in non-induced conditions led to reduced growth and might be the cause of the significantly reduced birth rate of YFP-BARD1δ transgenic mice as compared to non-transgenic littermates (Figure 2; Supplementary Figure S4).

Furthermore, we found both the upregulated expression of BARD1δ and telomere aberrations in cells from cancer patients with germline mutations in *BARD1* that predict expression of truncated FL BARD1 mRNAs (Figure 3).

It is widely accepted that FL BARD1 functions like BRCA1 in maintaining chromosomal stability and possibly telomere integrity [53, 55]. However, the repression of FL BARD1, as it is the case in many types of cancer, favors expression of BARD1δ. Our data suggest that human germline mutations predicting the reduced expression of FL BARD1 promote the upregulation of BARD1δ and lead to telomeric abnormalities.

This is in line with what is observed in most cancers: the tumor suppressor FL BARD1 is down-regulated, while the expression of other splice isoforms is boosted [14, 16–19]. The overexpression of BARD1δ, was found associated with all cancers investigated so far, but was particularly correlated with aggressive treatment resistant clear cell ovarian cancer [14]. Many studies suggest that the deficiency of FL BARD1 may have an oncogenic effect [11, 12, 16, 34, 39, 60]. However, lack of FL BARD1 associated with upregulated expression of isoforms is consistent with oncogenic functions attributed to alternatively spliced isoforms [8, 32, 46].

BARD1δ is derived from the splicing of exon 1 to 7 and lacks the RING domain, required for BRCA1 interaction, and the ANK repeats, required for p53 binding and apoptosis (Figure 1A) (13, 24). Thus, BARD1δ is deprived of tumor suppressor functions. BARD1δ was first identified as a BARD1 isoform upregulated in a rat cancer cell line NuTu-19 [13] which was derived from spontaneously transformed rat ovarian cells [61], suggesting this isoform of BARD1 and its cancer-associated functions are conserved between human and rat. It seems that the alternative splicing from exon 1 to 7 instead of from exon 1 to 2 has deleterious consequences. This was confirmed by the genome wide association study for neuroblastoma, where SNPs in intron 1 of *BARD1* were the most significantly associated with the disease (Carpasso et al., 2009). *BARD1* SNPs and mutations that affect splicing were also reported for breast and ovarian cancers [46, 47].

We found that both, BARD1δ and FL BARD1, bind to the components of the shelterin complex, namely TRF1, TRF2, and TNKS, which are essential for maintaining telomere integrity [42]. In contrast to FL BARD1, which does not significantly affect the function of shelterin, BARD1δ expression leads to depletion of shelterin components from telomere repeats. It was previously shown that such a depletion results in end to end chromosome fusions initiated by repair pathways [62]. In agreement with this, we demonstrate here that BARD1δ-dependend depletion of TRF1, TRF2, and TNKS from telomere repeats is associated with increased chromosomal aberrations and reduced cell proliferation. We suggest that BARD1δ antagonizes the chromosome and telomere protection function of FL BARD1 by sequestering shelterin complex components, thereby exposing the chromosomal ends to attrition and chromosome fusion and subsequent chromosomal instability.

In this study, we show that the “linker” region between the ANK and the BARD1 BRCT domains, which is retained in BARD1δ, can bind to novel BARD1-BRCA1 targets, such as TRF2 (Figure 5). The enhanced binding of BARD1δ to telomeric proteins depends on the particular structure of the “linker” in the context of BARD1δ, which is different from the structure in FL BARD1 or BARD1ω. Both contain ANK and “linker” sequences, but show reduced binding to telomeric proteins (Figure 5B, 5C) and do not induce telomere instability.

Thus, BARD1δ antagonizes FL BARD1-BRCA1 functions on key molecules that are important for chromosome integrity and proper segregation. While genomic instability induced by BARD1δ leads to cell cycle arrest in normal cells (Figure 1), in the absence of cell cycle control, e.g. due to p53 or pRB deficiencies, it enables cells with genomic instability to proliferate and acquire oncogenic functions. We therefore conclude that BARD1δ is a driver of cancer-associated genomic instability, thus providing the basis for carcinogenesis through the continued generation of genome alterations.

## MATERIALS AND METHODS

### Cell culture and transfection

Mammalian cells were cultured in RPMI medium (Invitrogen) supplied with fetal calf serum. The cells were transfected transiently (Figure 1) or stably with neomycin selection (Supplementary Figure S2) with plasmid DNA using X-tremeGENE 9 DNA Transfection Reagent (Roche), transfection reagent (QIAGEN).

### Expression constructs

FL BARD1, BARD1δ and BARD1ω biotin-tagged protein expression vectors: the corresponding protein coding DNA sequences were fused in-frame at the 3′end with the sequences encoding 23–amino acid *E.coli* BirA biotin ligase target [63] and inserted in the pcDNA3.1(+) vector. The resulting vectors were co-expressed with the pcDNA3.1-BirA construct at 10:1 ratio for recombinant protein biotin tagging.

EYFP expression constructs: pEYFP-C1 and pEYFP-C1 BARD1δ were described previously [38].

The expression plasmid for HA-tagged ubiquitin construct was described previously [33]

The pLSL-EYFP-BARD1δ construct for expression in transgenic mice was based on the pEYFP-C1-DELTA-RIN plasmid [38]. The β-globin intron from the pBS β-globin plasmid (gift from Pedro Herrera) was inserted upstream of the EYFP-BARD1δ fusion. The STOP element flanked by LoxP (LSL) [64] was inserted between CMV promoter and EYFP-BARD1δ coding sequence (see plasmid map in Supplementary Figure S3).

### Transgenic mice

Transgenic mice were generated by microinjection of the pLSL-EYFP-BARD1δ construct into the pronucleus of fertilized mouse eggs (line B6D2F1) and eggs were implanted in NMRI foster mothers. Transgenic offspring males were crossed to C57BL6J females to establish heterozygous C57BL/6J-Tg(LSL-YFP-BARD1δ) progeny. For CRE-dependent transgene induction C57BL/6J-Tg(LSL-YFP-BARD1δ) males were crossed to C57BL/6NTac -Gt(ROSA)26Sor^tm9(Cre/ESR1)Arte^ (Taconic) females. All animal experiments were performed at the Zootechnic facility of the University of Geneva with the authorization from the General Direction of Health, State of Geneva (No 1041/3685/3).

### Cell proliferation assay

HEK293 cells transfected with control or BARD1 expression plasmids were counted on a hemocytometer and then seeded with the same initial dilution in 6-well plates. Cell proliferation was monitored by counting the cells for 6 consecutive days.

### Flow cytometry

Flow cytometric analysis of DNA content was carried out by fluorescence-activated cell sorting (FACS) on Accuri C6 flow cytometer (BD Biosciences). The cells expressing biotinylated BARD1 proteins were stained with Streptavidin-DyLight488 (ThermoFisher Scientific), DAPI was used for DNA staining.

### Metaphase chromosome spreads and telomere FISH

Metaphase chromosome spreads were performed using standard protocols [65]. Telomere FISH was performed using DAKO Telomere PNA FISH Kit/Cy3 (DAKO). For the experiments involving human subjects informed consent was obtained from all patients, and the study was approved by the Medical Review Board of the Medical University of Gdansk, Poland (NKEB/399/2011-2012). The average number of each telomere aberration was calculated from at least 45 metaphases for each patient and 90 metaphases for healthy controls, or for a minimum of 25 metaphases for each mouse genotype.

### Immunofluorescent microscopy

Cultured cells were fixed with 2% paraformaldehyde and permeabilized with Triton X-100. Microscopy was performed using Nikon A1r spectral confocal system with Nikon NIS elements AR v4.20.01 64 bits software. The aperture of pinhole was optimized, the objective used was a Plan Apo lambda 60× oil, and, the pixel size (lateral resolution) was 0.12 microns/ pixel. These settings provide 0.47 micrometer optical section.

The antibodies used were BARD1 N19 (Santa Cruz), TRF1 (Santa Cruz), TRF2 (Santa Cruz), TNKS (ThermoFisher Scientific), β-tubulin (Sigma), fluorescent secondary antibodies (Life technologies), Streptavidin-DyLight488 (ThermoFisher Scientific).

### Immunoprecipitation and Western blotting

HEK293 cells were lysed in isotonic buffer with 1% Triton X-100. Lysate containing 400 μg of protein was incubated with 2 μg of antibody immobilized on protein G-Sepharose (Qiagen) over night at 4°C. The beads were washed with lysis buffer and proteins were eluted by heating at 95°C for 5 min in SDS loading buffer supplemented with 100 mM DTT.

Samples were subjected to SDS-PAGE and electro-blotted onto Immobilon-P (Millipore). Specific proteins were immuno-detected and visualized using enhanced chemiluminescence reagent (Amersham Biosciences) and X-ray film or GeneGnome chemiluminescence detection system (Syngene). The signal intensity was quantified using AlpfaEaseFC software (Alpha Innotech). For normalization of signals, Input or control signals from the same membrane were used. The antibodies used were BARD1 N19 (Santa Cruz), BARD1 H300 (Santa Cruz), TRF1 (Santa Cruz), TRF2 (Santa Cruz), TNKS (ThermoFisher Scientific), ABC biotin detection kit (ThermoFisher Scientific).

### Telomere chromatin immunoprecipitation (ChIP)

Transfected HEK293 cells were fixed for 4 min with 0.5% formaldehyde and lysed in RIPA buffer with 1% Triton X-100. Lysates were sonicated to obtain chromatin fragments of less than 1 kb. Lysate supernatants were incubated overnight with 2 μg of antibody immobilized on protein G-Sepharose (Qiagen) over night at 4°C. Pellets were washed and chromatin was eluted from the beads and crosslink was reversed. Hybridization with the biotin PCR-labeled TTAGGG probe or an Alu probe was performed and the biotinylated probe was detected with ABC kit (ThermoFisher Scientific). The intensity of the signal from telomere repeats was normalized to the signals of input DNA and Alu repeats. The antibodies used were TRF1 (Santa Cruz), TRF2 (Santa Cruz), TNKS (ThermoFisher Scientific), and GFP (Sigma).

### Structural modeling

Structural models for the isoforms of BARD1 were generated based on the experimental structures of the three globular domains: RING-type domain, (PDB: 1JM7), ANK repeats (PDB: 3C5R) and BRCT domains (PDB: 2R1Z). Structures were visualized with PyMOL (http://www.pymol.org/).

### RNA isolation and reverse transcription-PCR

Total RNA from cell lines and tissue specimens were extracted. For reverse transcription, we used M-MLV-Powerscript reverse transcriptase. PCR reactions were performed with Taq DNA polymerase. PCR products (15 μl) were analyzed on agarose/Tris-acetate-EDTA gels.

Irmgard Irminger-Finger has a conflict of interest. She is founder and shareholder of *BARD1 Life Sciences Limited*, a company developing BARD1-based biomarkers.

## SUPPLEMENTARY MATERIALS FIGURES AND TABLES


