# Supplementary material for: Antagonizing functions of BARD1 and its alternatively spliced variant BARD1δ in telomere stability
Source: Oncotarget. 2016 Dec 21;8(6):9339–53. doi: 10.18632/oncotarget.14068 (PMC5354735; doi:10.18632/oncotarget.14068)
Supplement: Supplementary file 1 [file oncotarget-08-9339-s001.pdf]

Antagonizing functions of BARD1 and its alternatively spliced variant BARD1δ in telomere stability

Supplementary Materials

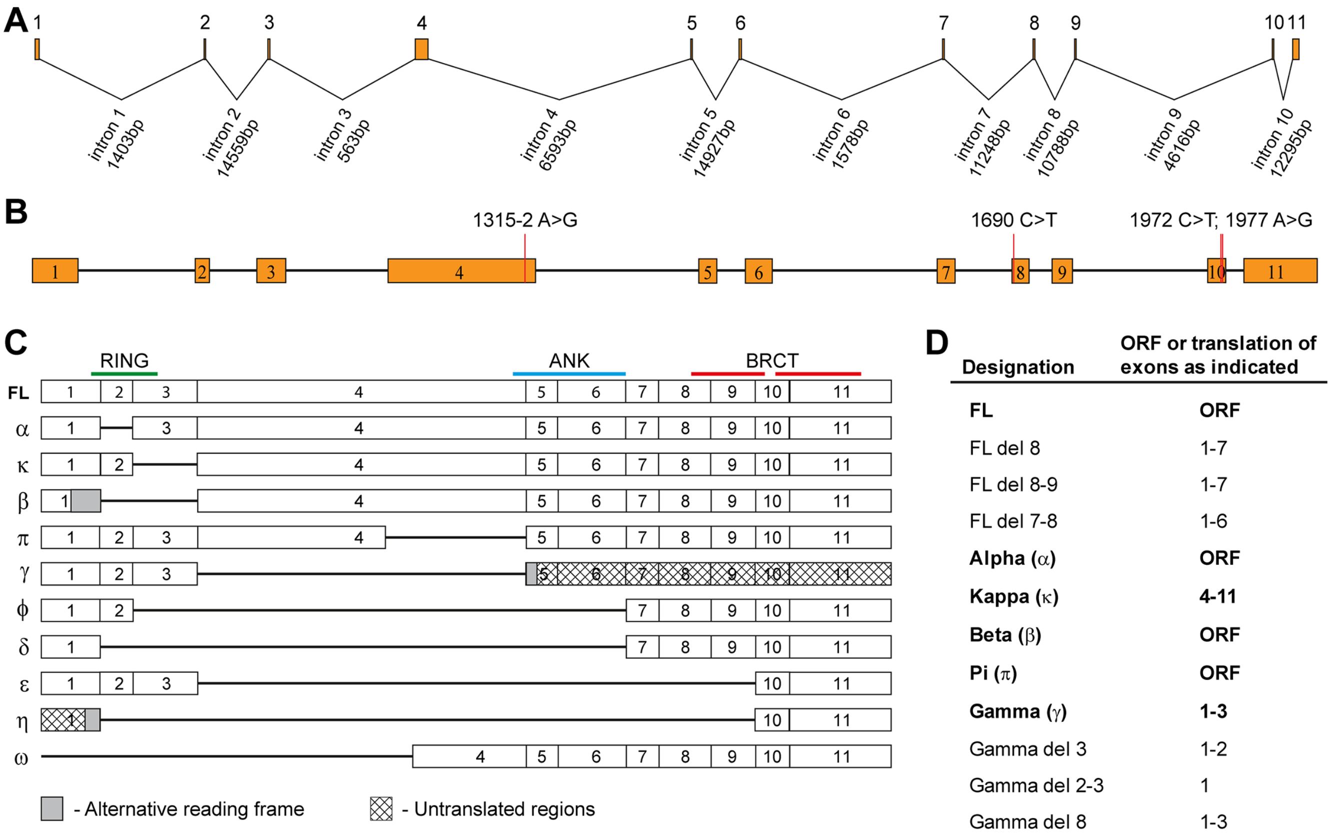

D

Designation	ORF or translation of exons as indicated
**FL**	**ORF**
FL del 8	1-7
FL del 8-9	1-7
FL del 7-8	1-6
**Alpha (α)**	**ORF**
**Kappa (κ)**	**4-11**
**Beta (β)**	**ORF**
**Pi (π)**	**ORF**
**Gamma (γ)**	**1-3**
Gamma del 3	1-2
Gamma del 2-3	1
Gamma del 8	1-3
Gamma del 2-3, 7	1
Gamma del 2-3, 8	1
Gamma del 5-7	1
Gamma del 2-3, 8-9	1
**Phi (φ)**	**ORF**
**Delta (δ)**	**ORF**
**Epsilon (ε)**	**ORF**
Epsilon del 3	1-2
**Eta (η)**	**ORF**
**Omega (ω)**	**ORF**

Supplementary Figure S1: BARD1 gene and its splice variants identified in cancer. (**A**) Schematic in scale representation of human BARD1 gene. The exon numbers are shown on the top, the intron numbers with the corresponding length in base pares are shown at the bottom. (**B**) Relative positions of human germ line mutations in BARD1 exons. The introns are shown not in scale. (**C**) The intron-exon structure of the BARD1 gene is shown with alignment of splice variants as first identified and designated in breast/ovarian cancer and lung cancer (Li et al., 2007; Zhang et al., 2012). (**D**) Compilation of mRNA isoforms identified in various cancers (Bosse et al., 2012; Sporn et al., 2011). The isoforms indicated in bold have an open reading frame (ORF) from the first ATG in exon 1 to stop in exon 11 and have been proven to be translated or are most likely translated (Bosse et al., 2012; Dizin and Irminger-Finger, 2010; Ryser et al., 2009). Isoforms with additional deletions resulting in premature termination are listed with designation plus indication of deleted exons (del x).

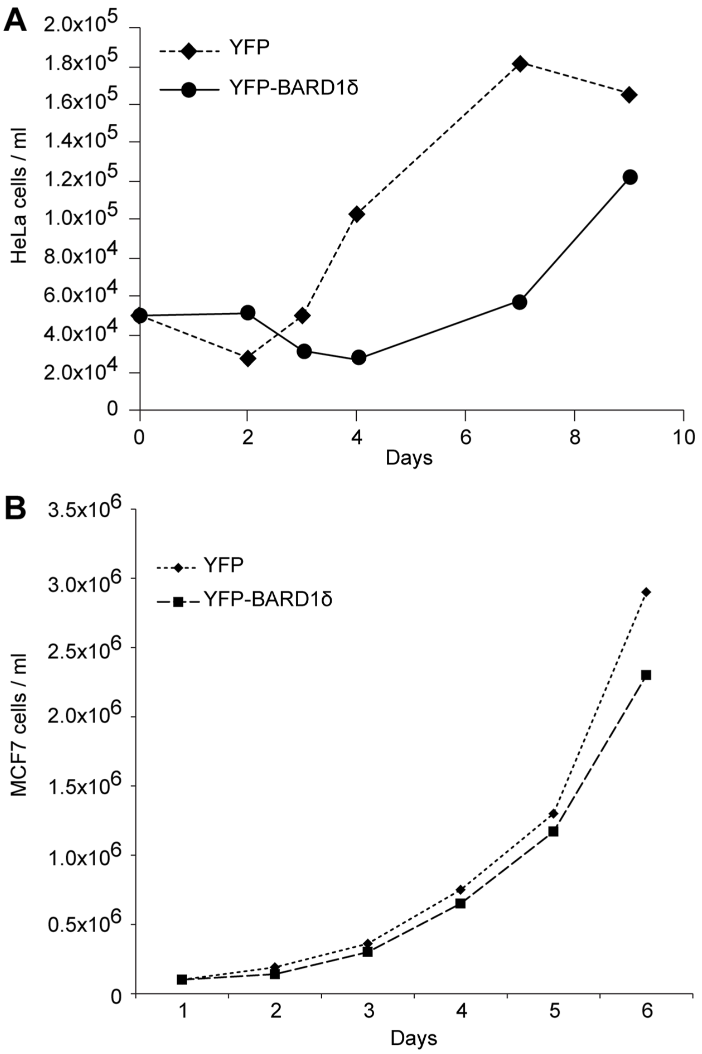

Supplementary Figure S2: BARD1δ reduces the growth rate of HeLa and MCF7 cells. (**A**) HeLa cells wre transfected with plasmids expressing YFP or YFP-BARD1δ, trypsinized and cell pellets were resuspended in 5 ml of D-MEM medium. The cells were counted on a hemocytometer and then seeded with the same initial dilution in 6-well plates. The resulting cultures were treated with neomycin to select for transfected cells. Cell growth was monitored by counting the cells for 9 consecutive days. BARD1δ overexpression repressed proliferation, but cells escaped from cell cycle block after several rounds of cell division, as observed for HEK 293 (Figure 2, main paper). (**B**) Growth curves of MCF7 cells transfected with YFP or YFP-BARD1δ. YFP-BARD1δ cells proliferation is reduced in comparison with control cells.

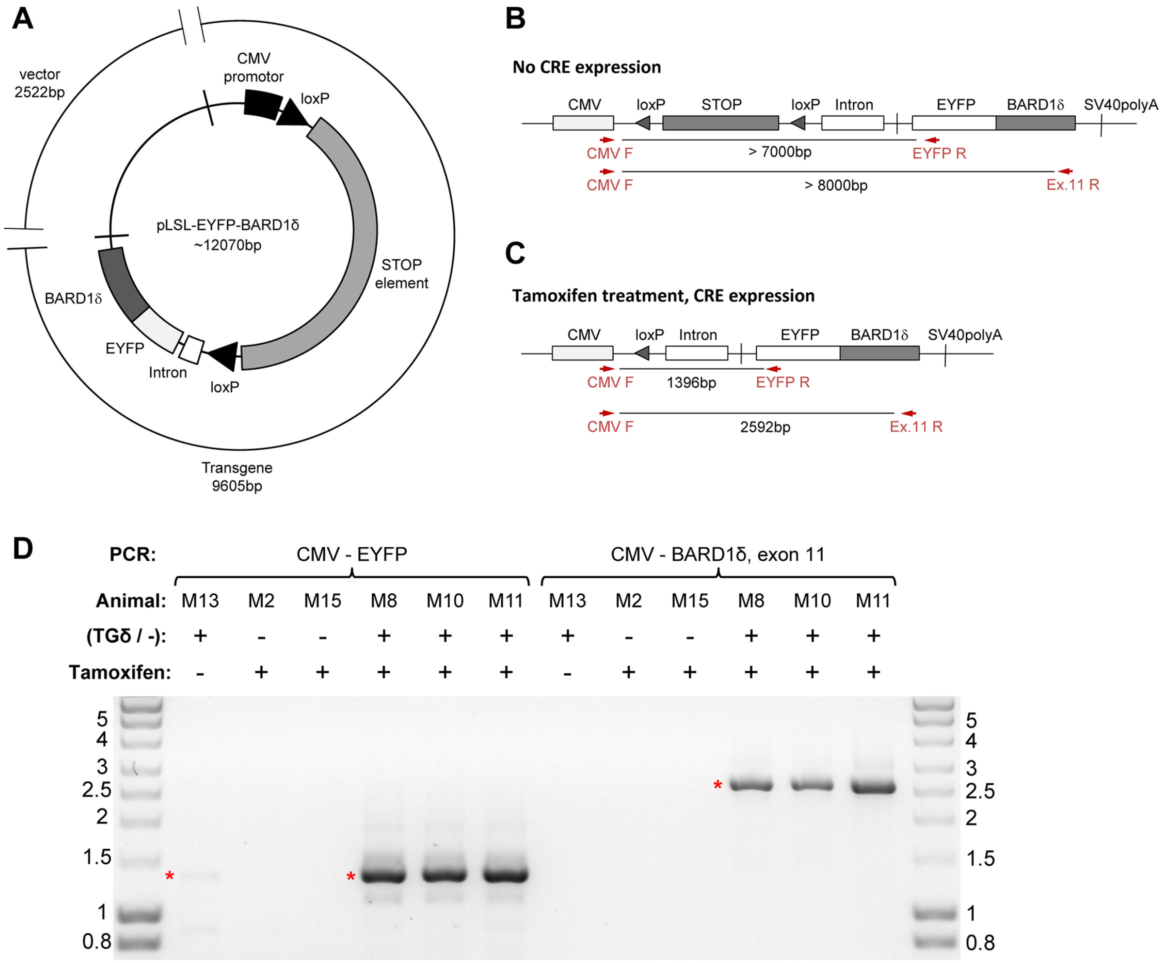

Supplementary Figure S3: Floxed EYFP-tagged BARD1δ for generation of transgenic mice. (**A**) The pLSL-EYFP-BARD1δ construct is based on the pEYFP-C1-DELTA-RIN plasmid (Tembe et Henderson, 2007). The β-globin intron from the pBS β-globin plasmid (gift from Pedro Herrera) was inserted upstream of the EGFP-BARD1δ fusion. The STOP element flanked by LoxP (Jackson et al, Genes Dev. 2001) was inserted between the CMV promotor and the EGFP-BARD1δ coding sequence. Fertilized mouse eggs were microinjected with the construct and transferred to foster mothers at the zoo-technology facilities at the University of Geneva. (**B**) Scheme of LSL-EYFP-BARD1δ construct without STOP element excision. Primers positions with expected fragment sizes are indicated. (**C**) Scheme of LSL-EYFP-BARD1δ construct after tamoxifen induced CRE excision of the STOP element. Primers positions with Expected fragment sizes are indicated. (**D**) PCR analzsis of genomic DNA from six randomly selected transgenic mice. All animals were Cre transgene heterozygous. The presence of BARD1δ transgene (TGδ) is indicated on the top with "+". The treatment with tamoxifen is indicated on the top with "+". Control vehicle treatment is indicated with "–". The PCR bands of expected size are indicated with asterisks. All three (TGδ / – ; Cre / –) mice treated with tamoxifen show the excision of STOP element and the presence of intact transgene. Note, that a marginally week signal corresponding to the excision of the STOP element (CMV-EYFP) is observed in M13 (TGδ / – ; Cre / –) mouse treated with vehicle. This may indicate the leakiness of CRE promoter. The fragments exceeding 7kb were not amplified.

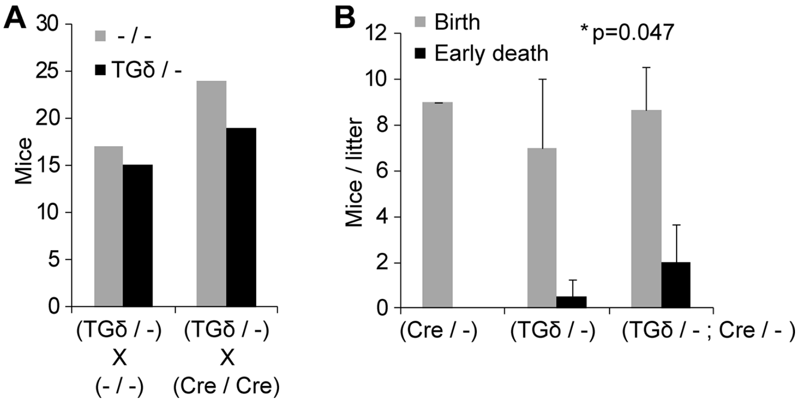

Supplementary Figure S4: BARD1δ transgenic mice birth rate and early mortality. (**A**) Number of heterozygote YFP-BARD1δ transgenic mice and mice not bearing YFP-BARD1δ transgene in the progeny of the cross of heterozygous YFP-BARD1δ transgenic mice (TGδ/–) to BL6 (–/–) or BL6 Cre mice (Cre/Cre). (**B**) Early mortality rate in the progeny of the cross of heterozygous YFP-BARD1δ transgenic mice (TGδ/–) to BL6 (–/–) or BL6 Cre mice (Cre/Cre).

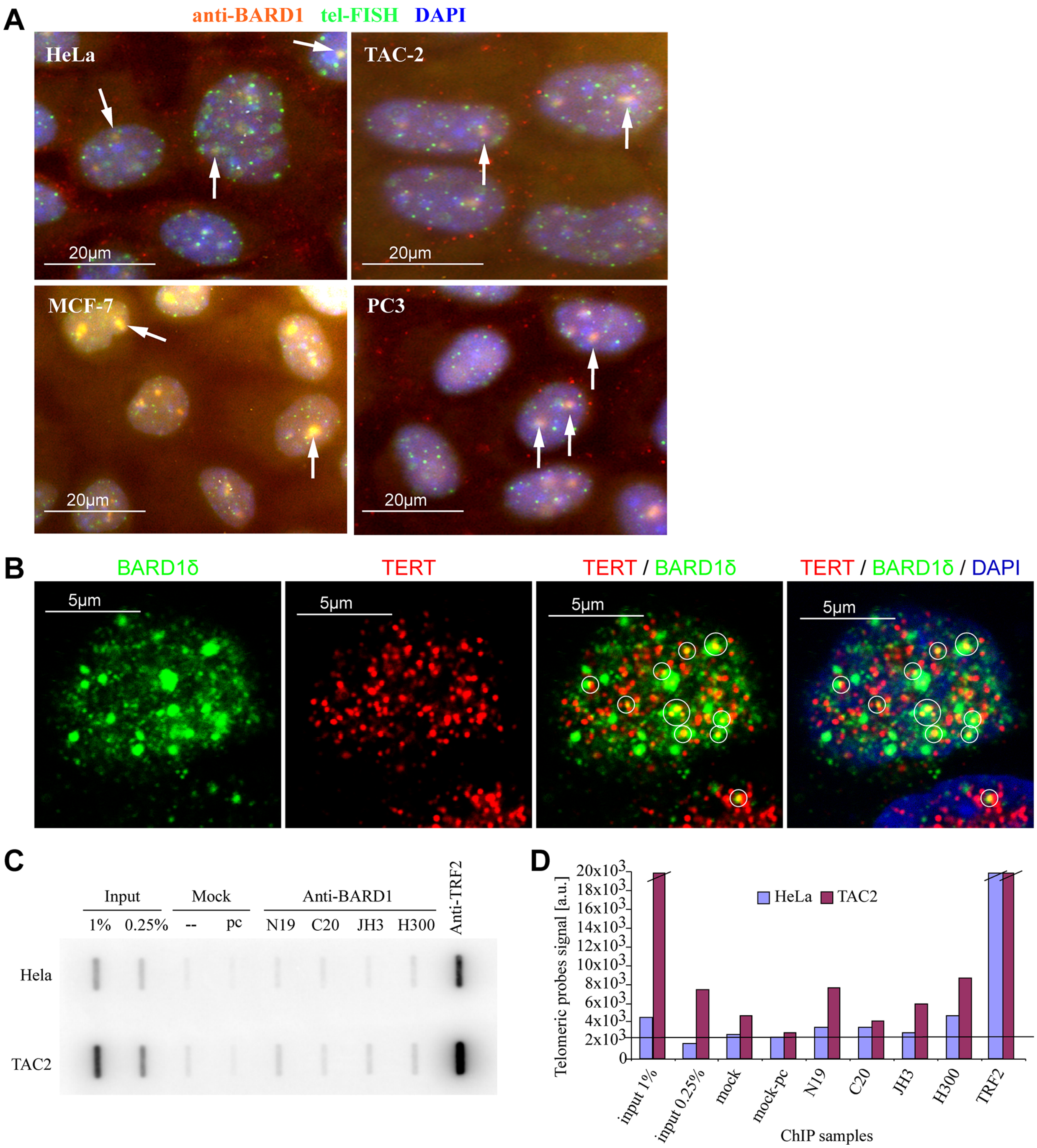

Supplementary Figure S5: BARD1 co-localizes with telomeres in different cell lines. (**A**) Triple staining with anti-BARD1 antibody (H300, Santa Cruz) for detection of full length BARD1, telomere-FISH (telFISH) and DAPI shows co-localization of BARD1 with telomeres in HeLa, MCF-7, PC3, and non-malignant mammary epithelial TAC2 cells (Irminger-Finger et al., 1998). (**B**) Co-localization of over-expressed FL BARD1 (green) and endogenous TERT (red) in HEK293 cells. Co-localization of FL BARD1 and TERT is marked with white circles. (**C**) Chromatin immunoprecipitation (ChIP) was performed from HeLa or TAC-2 cells with different anti-BARD1 antibodies: N19 (exon 1), C20 (exon 11), JH3 (exon 7), and H300 (exons 1–4). TRF2 antibodies were used as positive control. Co-precipitated DNA was blotted and probed for telomeric repeats. (**D**) Quantification of ChIP from panel C. All antibodies specifically precipitated telomere sequences from HeLa and TAC2 cells with similar efficacy, suggesting that FL BARD1 is binding to telomeres.

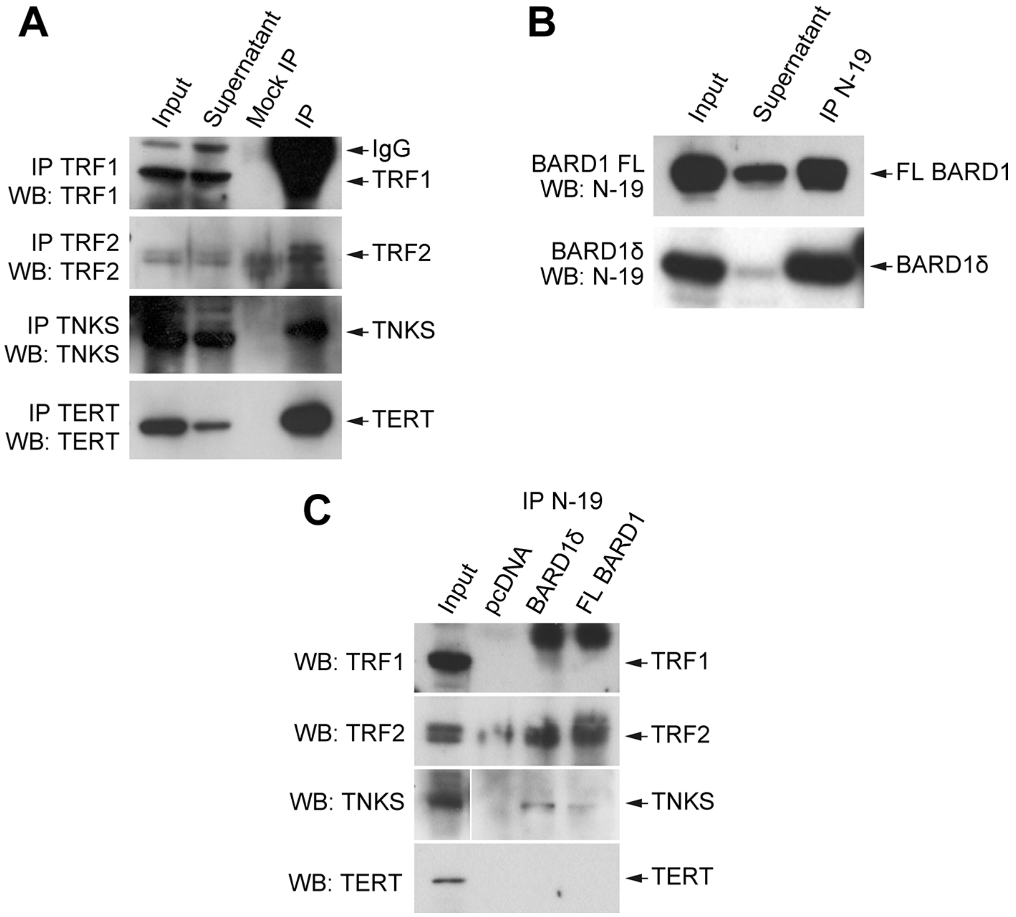

Supplementary Figure S6: Co-immunoprecipitation of FL BARD1 and BARD1δ with telomere binding proteins. (**A**) Western blot of the immunoprecipitation of endogenous TRF1, TRF2, TKNS, and TERT from HEK293 transfected with empty pcDNA vector. Input, supernatant, mock immunoprecipitation (IP), and IP fractions were loaded. Immunoprecipitated proteins and IgG are indicated with arrows (TRF2 appears as a double band). IPs of endogenous TRF1, TRF2, TKNS, and TERT from HEK293 cells overexpressing FL BARD1 and BARD1δ were performed identically in parallel (Figure 6, main paper). (**B**) Western blot of IP with anti-BARD1 N-19 antibody from HEK293 cells overexpressing FL BARD1 or BARD1δ. Input, supernatant, and IP fractions were loaded. Immunoprecipitated proteins are indicated with arrows. (**C**) Western blot of the co-IP of endogenous telomere proteins (TRF1, TRF2, TKNS, and TERT) with biotin-tagged FL BARD1 and BARD1δ overexpressed in HEK293 cells. The pcDNA control IP protein input, control IP from pcDNA transfected cells, and IPs from FL BARD1-bio and BARD1δ-bio extracts were loaded. Immunoprecipitated proteins were probed for TRF1, TRF2, TKNS, and TERT (TRF2 appears as a double band).

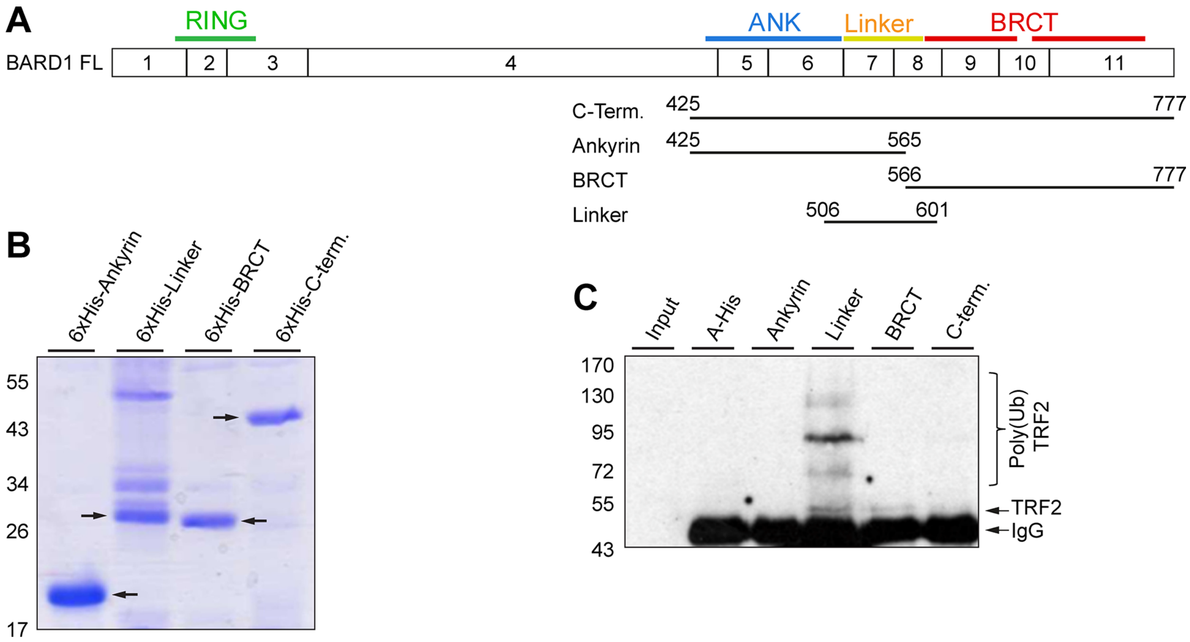

Supplementary Figure S7: BARD1 interaction studies. (**A**) Schematic presentation BARD1 exon structure aligned with His-tagged or GST fusion constructs of fragments of the BARD1 C-terminus. Relative positions of structural protein domains are indicated with RING, ANK, Linker, and BRCT. Amino acid positions are indicated to mark the extent of fusion proteins. (**B**) Coomassie stained SDS-PAGE gel of affinity purified BARD1 His or GST-fused polypeptides that were used for pull down assays of HEK293 cell extracts is shown. Arrows indicate respective BARD1 fusion proteins. (**C**) Pull-down assays using purified bacterially produced 6xHis-tagged BARD1 fragments. Tagged fusion proteins were incubated with whole cell extracts from HEK293 cells and pulled-down with anti-His antibodies. TRF2 binding was monitored on Western blot. TRF2 and IgG are indicated with arrows. Bracket indicates ubiquitinated forms of TRF2. Importantly, the whole BARD1 C-terminus had less binding activity than the Linker alone.

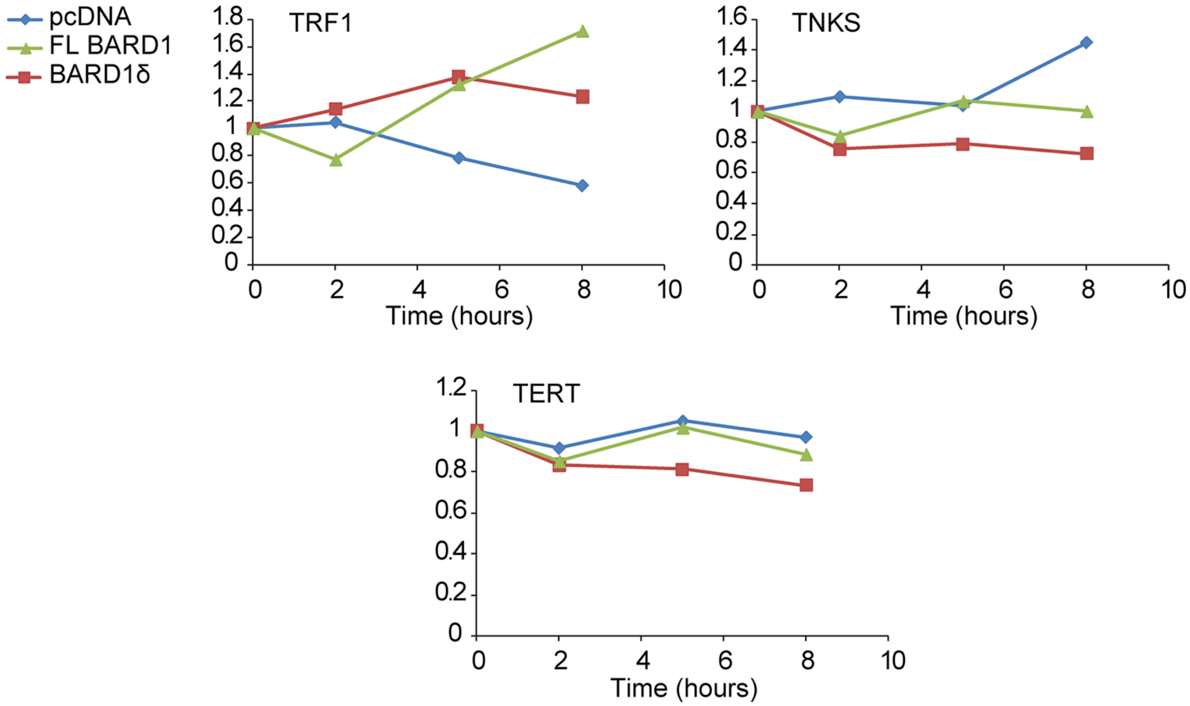

Supplementary Figure S8: FL BARD1 and BARD1δ affect stability of telomere binding proteins. The protein levels of TRF1, TNKS and TERT were measured by Western blot and subsequent densitometry in cells transfected with pcDNA control, FL BARD1, or BARD1δ at different time points of cycloheximide treatment. Signal intensities were normalized to actin intensity and to the intensity at zero time point.
